# An Integrative Polygenic and Epigenetic Risk Score for Overweight-related Hypertension in Chinese Population

**DOI:** 10.1093/gpbjnl/qzaf048

**Published:** 2025-06-16

**Authors:** Yaning Zhang, Qiwen Zheng, Qili Qian, Na Yuan, Tianzi Liu, Xingjian Gao, Xiu Fan, Youkun Bi, Guangju Ji, Peilin Jia, Sijia Wang, Fan Liu, Changqing Zeng

**Affiliations:** Henan Academy of Sciences, Zhengzhou 450046, China; China National Center for Bioinformation, Beijing 100101, China; Beijing Institute of Genomics, Chinese Academy of Sciences, Beijing 100101, China; CAS Key Laboratory of Computational Biology, Shanghai Institute of Nutrition and Health, Chinese Academy of Sciences, Shanghai 200031, China; University of Chinese Academy of Sciences, Beijing 100049, China; China National Center for Bioinformation, Beijing 100101, China; Beijing Institute of Genomics, Chinese Academy of Sciences, Beijing 100101, China; CAS Key Laboratory of Computational Biology, Shanghai Institute of Nutrition and Health, Chinese Academy of Sciences, Shanghai 200031, China; China National Center for Bioinformation, Beijing 100101, China; Beijing Institute of Genomics, Chinese Academy of Sciences, Beijing 100101, China; National Clinical Research Center of Kidney Diseases, Jinling Hospital, Nanjing 210002, China; China National Center for Bioinformation, Beijing 100101, China; Beijing Institute of Genomics, Chinese Academy of Sciences, Beijing 100101, China; University of Chinese Academy of Sciences, Beijing 100049, China; Institute of Biophysics, Chinese Academy of Sciences, Beijing 100101, China; Henan Academy of Sciences, Zhengzhou 450046, China; China National Center for Bioinformation, Beijing 100101, China; Beijing Institute of Genomics, Chinese Academy of Sciences, Beijing 100101, China; University of Chinese Academy of Sciences, Beijing 100049, China; CAS Key Laboratory of Computational Biology, Shanghai Institute of Nutrition and Health, Chinese Academy of Sciences, Shanghai 200031, China; University of Chinese Academy of Sciences, Beijing 100049, China; Department of Forensic Sciences, College of Criminal Justice, Naif Arab University for Security Sciences, Riyadh 12271, Saudi Arabia; Henan Academy of Sciences, Zhengzhou 450046, China; China National Center for Bioinformation, Beijing 100101, China; Beijing Institute of Genomics, Chinese Academy of Sciences, Beijing 100101, China; University of Chinese Academy of Sciences, Beijing 100049, China

**Keywords:** Overweight-related hypertension, Polygenic risk score, Methylation risk score, Multi-omics prediction, Academic

## Abstract

Overweight-related hypertension (OrH), defined by the coexistence of excess body weight and hypertension (HTN), is an increasing health concern elevating cardiovascular disease risks. In this study, we evaluated the prediction performance of polygenic risk scores (PRSs) and methylation risk scores (MRSs) for OrH in 7605 Chinese participants from two cohorts: the Chinese Academy of Sciences (CAS) and the National Survey of Physical Traits (NSPT). In the CAS cohort, which predominantly consists of academics, males showed significantly higher prevalence of obesity, HTN, and OrH, along with worse metabolic syndrome indicators, compared to females. This disparity was less pronounced in the NSPT cohort and in broader Chinese epidemiological studies. Among ten PRS methods, PRS-CSx was the most effective, enhancing prediction accuracy for obesity [area under the curve (AUC) = 0.75], HTN (AUC = 0.74), and OrH (AUC = 0.75), compared to baseline models using only age and sex (AUC = 0.55–0.71). Similarly, least absolute shrinkage and selection operator (LASSO)-based MRS models improved prediction accuracy for obesity (AUC = 0.70), HTN (AUC = 0.73), and OrH (AUC = 0.78). Combining PRS and MRS further boosted prediction accuracy, achieving AUC values of 0.77, 0.76, and 0.80 for obesity, HTN, and OrH, respectively. These models stratified individuals into high (> 0.6) or low (< 0.1) risk categories, covering 59.95% for obesity, 31.75% for HTN, and 43.89% for OrH. Our findings highlight a higher OrH risk among male academics, emphasize the influence of metabolic and lifestyle factors on MRS predictions, and highlight the value of multi-omics approaches in enhancing risk stratification.

## Graphical abstract

**Figure:**
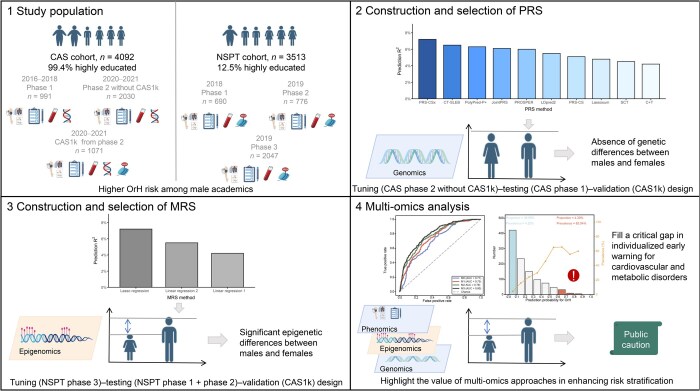

## Introduction

Overweight-related hypertension (OrH) is a distinct clinical condition characterized by the concurrent disorders of both body weight and blood pressure [1–3]. Its global prevalence has largely increased over the past two decades, linking to the rising risks of cardiovascular and cerebrovascular diseases [4,5]. Recent genome-wide association studies (GWAS) [6] and epigenome-wide association studies (EWAS) [7] have uncovered numerous genetic and epigenetic factors associated with body weight and blood pressure. To date, the National Human Genome Research Institute - European Bioinformatics Institute (NHGRI-EBI) GWAS catalog [8,9] has cataloged 4263 single nucleotide polymorphisms (SNPs) from 54 studies that are significantly associated with body mass index (BMI), spanning 1252 genes. Meanwhile, 36 studies have identified 2853 SNPs across 862 genes significantly associated with diastolic blood pressure (DBP) and systolic blood pressure (SBP) (Table S1). Furthermore, EWAS have identified 1581 CpG sites across 855 genes that are associated with BMI [10–15] along with 150 CpG sites from 85 genes associated with blood pressure (Table S2) [16–22].

With the continuous discovery of a large number of genetic and epigenetic risk factors, polygenic risk scores (PRSs) [23,24] and methylation risk scores (MRSs) [18,25] have emerged as pivotal tools for profiling the risk landscape of OrH. For instance, a meta-analysis involving 700,000 European individuals constructed a PRS using 941 SNPs, which explained approximately 6% of the variance in BMI [26]. A stratification analysis from the Korean Genome and Epidemiology Study (KoGES) showed that participants in the highest PRS quartile had a two-fold increased risk of obesity and hypertension (HTN) compared to those in the lowest quartile [27]. Similarly, using EWAS data of nearly 5000 Europeans and Africans, a MRS constructed from 33 CpG loci accounted for 3.31% and 3.99% of the variance in SBP and DBP, respectively [28]. Additionally, a MRS based on 435 CpG sites, derived from penalized regression of methylation data from 2562 unrelated participants in Generation Scotland, explained around 10% of BMI variance, with each standard deviation (SD) increase in MRS associated with a 37% higher risk of obesity [29]. Furthermore, by contrasting MRS with PRS, a recent review emphasized the importance of integrating genetic and epigenetic data for improved trait prediction [25]. Indeed, studies combining PRS and MRS have demonstrated an increase in the explained variance in BMI, up to 14% [30] and 19% [10], highlighting the potential of multi-omics approaches.

Despite these achievements, the prediction of OrH, a comorbidity with various disorders, remains underexplored. The construction of PRS and MRS for OrH faces several challenges, especially in the Chinese population. One major limitation is the reduced efficacy of PRS and MRS when developed in one ancestry group and applied to others. To date, well-powered GWAS and EWAS have predominantly focused on individuals of European ancestry, limiting their applicability to other populations [31,32]. Moreover, cultural and environmental factors unique to the Chinese population may influence how genetic variations and epigenetic modifications contribute to disease risk [7,33].

On the other hand, multiple approaches have been developed for constructing PRS and MRS. In addition to the classic clumping and thresholding (C+T) method [34], shrinkage methods, such as Stacked C+T (SCT) [35], PRS-CS [36], LDpred2 [37,38], and lassosum [39], adjust the weight of SNPs based on linkage disequilibrium (LD) information. Other methods, such as PRS-CSx [40], CT-SLEB [41], PolyPred-P+ [42], JointPRS [43], and Polygenic Risk scOres based on an enSemble PEnalized Regression (PROSPER) [44], are specifically designed for deployment across multiple ancestries, enhancing generalizability across diverse populations. For MRS construction, common computational approaches include C+T [30] and penalized linear regression [11]. Furthermore, effectively integrating PRS and MRS could offer a promising opportunity to improve risk prediction for OrH.

In an attempt to accurately assess OrH in the Chinese population, this study aimed to construct an integrative multi-omics model. Using data from 3021 individuals in the Chinese Academy of Sciences (CAS) cohort, we evaluated various PRS methodologies based on GWAS statistics from the BioBank Japan (BBJ) and the UK Biobank (UKB). Simultaneously, we analyzed several MRS models based on prior EWAS findings using data from 3513 individuals in the National Survey of Physical Traits (NSPT) cohort. The performance of both PRS and MRS in predicting OrH risk was further validated in a separate dataset of 1071 individuals from the CAS cohort.

## Results

### Higher obesity, HTN, and OrH risk in male academics

Our study included a total of 7605 Chinese individuals from two cohorts: 991 participants of phase 1 and 3101 of phase 2 from the CAS cohort with phenotypic and genomic data, and 3513 participants from the NSPT cohort with phenotypic and DNA methylation data. Additionally, methylation data were available for 1071 samples in phase 2 of the CAS cohort. According to the baseline data in **Table 1**, both the CAS and NSPT cohorts are middle-aged (average age: 39.42 ± 10.11 years and 50.21 ± 12.75 years, respectively), with slightly fewer males (47.8% and 37.1%, respectively). A notable feature of the CAS cohort is the high proportion of participants with higher education (99.4% compared to 12.5% in the NSPT cohort).

**Table 1:** Baseline characteristics of participants in the study

	Characteristic	Total	Obesity (BMI ≥ 28)			HTN (SBP ≥ 140 or DBP ≥ 90)			OrH (BMI ≥ 24 with HTN)		
			Control	Case	*P*	Control	Case	*P*	Control	Case	*P*
CAS cohort	Number	4092	3724	368		3314	778		3572	520	
	Age (year): mean ± SD	39.42 ± 10.11	39.18 ± 10.10	41.81 ± 9.93	5.76E−06	38.05 ± 9.35	45.27 ± 11.10	6.18E−59	38.42 ± 9.63	46.27 ± 10.69	2.20E−54
	Male: number (%)	1955 (47.78%)	1669 (44.82%)	286 (77.72%)	3.60E−33	1388 (41.88%)	567 (72.88%)	2.52E−72	1534 (42.95%)	421 (80.96%)	8.50E−59
	Higher education: number (%)	4067 (99.39%)	3700 (99.36%)	367 (99.73%)	0.60	3303 (99.67%)	764 (98.20%)	0.12	3554 (99.50%)	513 (98.65%)	0.05
	SBP (mmHg): mean ± SD	118.17 ± 14.26	117.10 ± 13.69	129.05 ± 15.34	1.08E−48	114.03 ± 10.78	135.84 ± 13.78	1.22E−256	115.34 ± 11.99	137.63 ± 13.41	7.13E−206
	DBP (mmHg): mean ± SD	76.57 ± 10.95	75.83 ± 10.56	84.12 ± 11.94	6.65E−42	73.35 ± 8.48	90.31 ± 9.59	2.22E−303	74.43 ± 9.44	91.26 ± 9.24	3.69E−234
	BMI (kg/m^2^): mean ± SD	23.80 ± 3.26	23.14 ± 2.44	30.48 ± 3.01	0	23.34 ± 3.05	25.75 ± 3.40	2.12E−68	23.27 ± 2.98	27.42 ± 2.78	1.64E−157
NSPT cohort	Number	3513	2970	543		2314	1199		2741	782	
	Age (year): mean ± SD	50.21 ± 12.75	50.34 ± 13.01	49.49 ± 11.21	0.20	47.26 ± 13.03	55.89 ± 9.99	1.03E−52	50.03 ± 12.95	52.40±9.75	5.07E−03
	Male: number (%)	1304 (37.12%)	1064 (35.82%)	240 (44.20%)	2.47E−04	783 (33.84%)	521 (43.45%)	2.75E−08	957 (34.91%)	353 (45.14%)	2.25E−07
	Higher education: number (%)	439 (12.49%)	372 (12.53%)	67 (12.34%)	0.96	391 (16.90%)	48 (4.00%)	1.10E−27	403 (12.42%)	37 (4.73%)	1.57E−13
	SBP (mmHg): mean ± SD	129.47 ± 20.41	128.24 ± 20.43	136.17 ± 18.98	2.26E−14	118.16 ± 11.26	151.29 ± 15.91	3.55E−284	127.71 ± 19.81	150.88 ± 14.70	1.32E−62
	DBP (mmHg): mean ± SD	80.65 ± 11.79	79.68 ± 11.50	85.98 ± 11.91	5.92E−26	75.02 ± 8.27	91.54 ± 9.77	2.63E−224	79.57 ± 11.22	93.87 ± 10.47	1.50E−74
	BMI (kg/m^2^): mean ± SD	24.49 ± 3.54	23.44 ± 2.62	30.24 ± 2.12	0	23.97 ± 3.41	25.50 ± 3.59	3.76E−23	24.00 ± 3.16	30.52 ± 2.11	9.31E−182

*Note*: Characteristics of participants in the CAS cohort (*n* = 4092) and the NSPT cohort (*n* = 3513), stratified by HTN, obesity, and OrH, are presented. The table includes the distribution of higher education, age, sex, SBP, DBP, and BMI for both control and case groups. Data are presented as mean ± SD and number (%) for characteristics. Statistical significance (*P*) for differences between cases and controls is indicated. The self-reported clinically diagnosed HTN or the use of antihypertensive medication were included as HTN cases. BMI, body mass index; DBP, diastolic blood pressure; SBP, systolic blood pressure; HTN, hypertension; OrH, overweight-related hypertension; CAS cohort, Chinese Academy of Sciences cohort; NSPT cohort, National Survey of Physical Traits cohort; SD, standard deviation.

**Figure 1:**
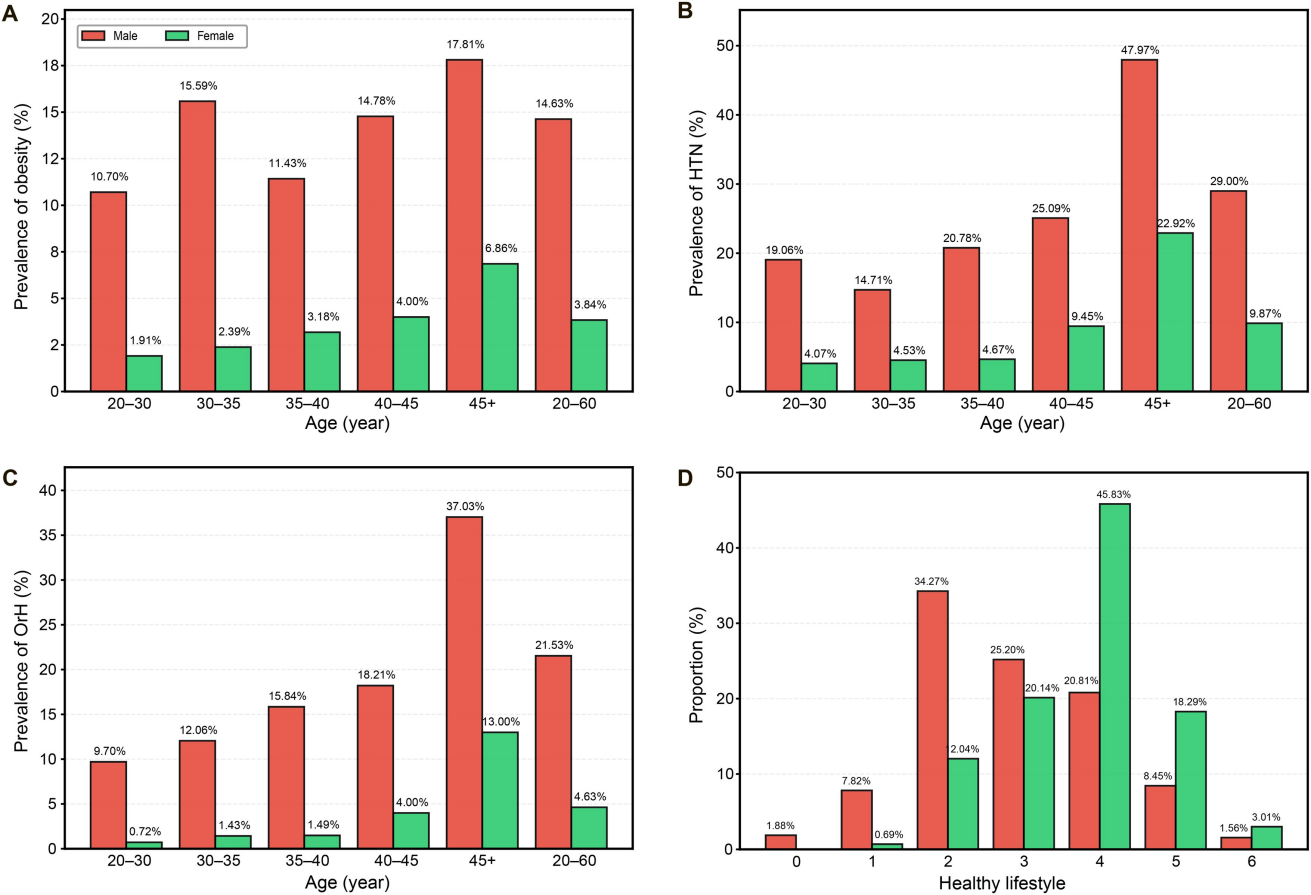
Prevalence of obesity, HTN, and OrH across age groups and healthy lifestyle scores by gender **A**.–**C**. Prevalence of obesity (A), HTN (B), and OrH (C) in males (red) and females (green) across different age groups in the CAS cohort (*n* = 4092). **D**. Distribution of healthy lifestyle scores by gender in the CAS1k cohort (*n* = 1071). The healthy lifestyle score, ranging from 0 (least healthy) to 6 (most healthy), is calculated as the sum of six binary criteria: non-smoking, no excessive alcohol consumption, healthy diet (daily intake of fruits and vegetables), regular physical activity, normal BMI (18.5–23.9 kg/m^2^), and normal WC (< 85 cm for males and < 80 cm for females). BMI, body mass index; HTN, hypertension; OrH, overweight-related hypertension; WC, waist circumference; CAS cohort, Chinese Academy of Sciences cohort.

A distinct pattern was observed in the CAS cohort, where obesity, HTN, and OrH exhibited notably higher male-to-female prevalence ratios (M/F ratios) compared to both the NSPT cohort and broader Chinese epidemiological studies. In the CAS cohort, the proportion of males was 77.72% in obesity cases, 72.88% in HTN cases, and 80.96% in OrH cases (Table 1). This corresponded to M/F ratios of 3.8 (14.63% *vs.* 3.84%, *P* = 3.60E−33) for obesity, 2.9 (29.00% *vs.* 9.87%, *P* = 2.52E−72) for HTN, and a particularly concerning 4.7 (21.53% *vs.* 4.63%, *P* = 8.50E−59) for OrH. Notably, this gender disparity persisted across all age groups (**Figure 1**). In contrast, the proportions of males in obesity, HTN, and OrH cases were all lower in the NSPT cohort (44.20%, 43.45%, and 45.14%, respectively), with the gender disparity (M/F ratio) being much less pronounced (1.34, 1.30, and 1.40, respectively). The M/F ratio of the NSPT cohort was similar to that observed in the national survey on obesity and HTN (Table S3) [45–50].

Additionally, compared to the NSPT cohort, gender disparities in metabolic health were more pronounced in the CAS cohort. CAS males exhibited significantly worse levels of multiple metabolic syndrome indicators, including total cholesterol (TC), triglycerides (TG), low-density lipoprotein (LDL), and high-density lipoprotein (HDL) (Table S4). In contrast, CAS females demonstrated significantly better indicators, including TG, HDL, LDL, and fasting blood glucose (FBG), compared to NSPT females. These findings highlight a notable gender difference in the health conditions of academics in China.

Furthermore, we assessed healthy lifestyle scores in the CAS cohort, calculated as the sum of six binary indicators (Table S5). As demonstrated in Figure 1, as high as 67.1% of women had a score of ≥ 4, compared to only 30.8% of men who reached this threshold (*P* = 2.54E−31). Normal BMI (18.5–23.9 kg/m^2^) and waist circumference (WC; < 85 cm for men and < 80 cm for women) showed the most notable gender differences: normal BMI (34.43% in men *vs*. 66.20% in women, *P* = 3.12E−24) and normal WC (35.52% in men *vs*. 74.31% in women, *P* = 2.84E−35). Significant differences were also observed in the absence of current smoking (81.06% in men *vs*. 99.31% in women, *P* = 1.36E−19) and the absence of excessive drinking (92.64% in men *vs*. 98.84% in women, *P* = 7.30E−06).

### PRS-CSx outperforms other PRS methods

In this analysis, we utilized subsets from the CAS cohort for PRS tuning, testing, and validation (see Materials and methods), including: the PRS tuning set (*n* = 2030, phase 2 without CAS1k), the PRS testing set (*n* = 991, phase 1), and the validation set (*n* = 1071, CAS1k). For quantitative traits including BMI, DBP, and SBP, 10 PRS methods (C+T, SCT, PRS-CS, LDpred2, lassosum, PRS-CSx, CT-SLEB, PolyPred-P+, JointPRS, and PROSPER) were trained on the PRS tuning set using GWAS summary statistics from the UKB (*n* ≈ 450,000, European) and the BBJ (*n* ≈ 150,000, Japanese) (Figures S1 and S2; Table S6). The latter five methods (PRS-CSx, CT-SLEB, PolyPred-P+, JointPRS, and PROSPER) represent multi-ancestry PRSs, where weights were derived by integrating UKB and BBJ GWAS data.

**Figure 2:**
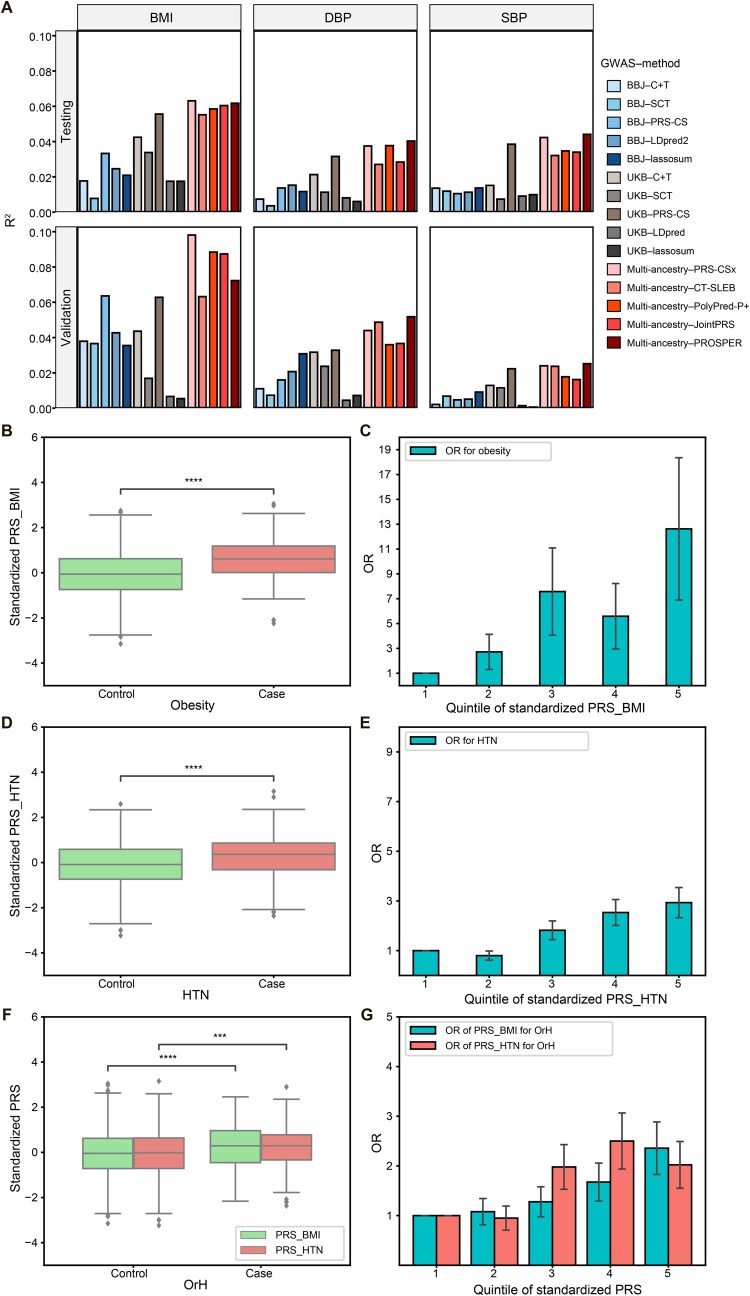
Performance of PRSs for BMI, DBP, and SBP as well as their associations with obesity, HTN, and OrH **A**. R^2^ values (Y-axis) represent the proportion of phenotypic variance explained by the PRSs for BMI, DBP, and SBP across different GWAS–method combinations (X-axis) in the testing set (*n* = 991, upper) and the validation set (*n* = 1071, lower). Each bar corresponds to a specific method, as indicated by the color-coded legend. The phenotype was regressed on age, sex, and six principal components, and the residual from this regression was used as the dependent variable in the PRS modeling analyses. **B**. Boxplot of PRS for BMI (PRS_BMI) in control *vs*. obesity case groups. **C**. OR for obesity across quintiles of PRS_BMI, with the lowest 20% quintile serving as the reference group. **D**. Boxplot of PRS for HTN (PRS_HTN) in control *vs*. HTN case groups. **E**. OR for HTN across quintiles of PRS_HTN, with the lowest 20% quintile serving as the reference group. **F**. Boxplot of PRS for BMI (PRS_BMI) and HTN (PRS_HTN) in control *vs*. OrH case groups. **G**. OR for OrH across quintiles of PRS_BMI and PRS_HTN, with the lowest 20% quintile serving as the reference group. ****, *P* < 0.0001; ***, *P* < 0.001 (Wald test). DBP, diastolic blood pressure; SBP, systolic blood pressure; PRS, polygenic risk score; OR, odds ratio; GWAS, genome-wide association study; C+T, clumping and thresholding; SCT, Stacked C+T; PROSPER, Polygenic Risk scOres based on an enSemble PEnalized Regression; UKB, UK Biobank; BBJ, BioBank Japan.

Overall, throughout the tuning–testing–validation process (Tables S7–S12), the PRS generated by PRS-CSx demonstrated robust performance, showing relatively strong prediction ability for the residuals of quantitative phenotypes (including BMI, SBP, and DBP) after regressing out age, sex, and six genomic principal components. Specifically, PRS-CSx achieved an R^2^ of 2.40%–9.81% in predicting residual variance, with an average of 4.76% in the testing set (slightly lower than 4.87% for PROSPER) and 5.54% in the validation set (higher than 4.98% for PROSPER) (**Figure 2**A; Table S12).

When compared to the PRSs from the Polygenic Score (PGS) Catalog, the PRS-CSx method showed strong performance across multiple traits (BMI, SBP, and DBP) in both testing and validation sets (Table S13). The exceptions were for DBP in the testing set, where PRS-CSx ranked second, slightly lower than PGS003964 (3.75% *vs*. 4.90%), and for SBP, where PRS-CSx ranked second in the testing set and third in the validation set, with marginal differences from the top-performing PRS. These results underscore the broad applicability and robustness of PRS-CSx in capturing the genetic architecture of complex traits, leading us to choose PRS-CSx for subsequent analyses.

### Prediction accuracy of PRS-CSx for quantitative and binary traits

After comparison and selection of PRS-CSx, we further assessed its prediction performance for quantitative traits (BMI, SBP, and DBP) and binary disease outcomes (obesity, HTN, and OrH) in the validation set (*n* = 1071, CAS1k). The PRSs were approximately normally distributed (Kolmogorov–Smirnov normality test with Bonferroni correction for multiple comparisons, *P* > 0.05).

For quantitative traits, the baseline models, which incorporated sex and age only, yielded R^2^ values of 18.28% for BMI, 17.86% for DBP, and 20.47% for SBP. When these models were augmented with respective PRS-CSx, there was a significant increase in accuracy, evidenced by R^2^ values of 26.54% for BMI, 21.21% for DBP, and 22.71% for SBP (**Table 2**).

**Table 2:** Performance of different models for obesity, HTN, and OrH in the validation set

Model	R^2^ (95% CI)			AUC (95% CI)		
	BMI	SBP	DBP	Obesity	HTN	OrH
Baseline model	18.28% (13.79%–22.48%)	20.47% (16.02%–24.63%)	17.86% (13.69%–21.84%)	0.55 (0.51–0.59)	0.70 (0.66–0.73)	0.71 (0.68–0.75)
PRS model	26.54% (21.71%–31.01%)	22.71% (18.10%–26.81%)	21.21% (16.51%–25.31%)	0.75 (0.70–0.79)	0.74 (0.71–0.77)	0.75 (0.72–0.78)
MRS model	26.86% (22.52%–31.00%)	25.72% (20.87%–29.89%)	23.15% (18.42%–27.42%)	0.70 (0.65–0.74)	0.73 (0.69–0.76)	0.78 (0.74–0.81)
Multi-omics model	33.98% (29.12%–38.52%)	27.58% (22.76%–31.61%)	26.02% (21.42%–30.61%)	0.77 (0.72–0.81)	0.76 (0.73–0.79)	0.80 (0.77–0.83)

*Note*: The performance of baseline model (only with age and sex), PRS model (with age, sex, and PRS), MRS model (with age, sex, and MRS), and multi-omics model (with age, sex, PRS, and MRS) was evaluated using R^2^ for BMI, DBP, and SBP and AUC for obesity, HTN, and OrH. All metrics and 95% CIs were determined using a five-fold cross-validation approach in the validation set (*n* = 1071). PRS, polygenic risk score; MRS, methylation risk score; AUC, area under the curve; CI, confidence interval.

Similarly, for binary disease statuses including obesity, HTN, and OrH, models integrated with PRS-CSx also demonstrated improved accuracy compared to the baseline models. PRS_BMI distinctively segregated obesity from the non-obesity group (*P* = 4.35E−11, Figure 2B), with an odds ratio (OR) for obesity increased by 2.13 for each SD increment (OR/SD) in PRS_BMI [95% confidence interval (CI): 1.77–2.57, *P* = 1.70E−11] (Table S14). In a five-quantile schema, the OR for obesity rose progressively across quintiles, reaching 12.63 in the highest quintile (Figure 2C; Table S14). This inclusion of PRS_BMI notably enhanced the model’s accuracy for obesity prediction, increasing the area under the curve (AUC) from 0.55 to 0.75 (Table 2). For HTN, the combined PRS_SBP and PRS_DBP (PRS_HTN) significantly differentiated between the HTN and non-HTN groups (*P* = 2.24E−08) (Figure 2D), with an OR/SD of 1.68 in PRS_HTN (95% CI: 1.47–1.91, *P* = 1.04E−10) (Table S14). The OR for HTN increased gradually across the five quantiles, peaking at 2.93 in the highest quintile (Figure 2E; Table S14). The inclusion of PRS_HTN in the model resulted in an improvement in prediction accuracy for HTN, increasing AUC from 0.70 to 0.74 (Table 2). Lastly, the model for predicting OrH showed a significant improvement in accuracy when including both PRS_BMI and PRS_HTN as predictors, with the AUC increasing from 0.71 to 0.75 (Table 2). Both PRS_BMI and PRS_HTN significantly differentiated between the OrH and non-OrH groups (*P* = 7.72E−05 and *P* = 5.16E−04, respectively) (Figure 2F), with an OR/SD of 1.42 in PRS_BMI (95% CI: 1.23–1.63, *P* = 3.88E−05) (Table S14) and 1.45 in PRS_HTN (95% CI: 1.26–1.66, *P* = 1.63E−05) (Table S14). In the highest quintile, the ORs for OrH peaked at 2.36 and 2.02 for PRS_BMI and PRS_HTN, respectively (Figure 2G; Table S14).

We further compared gender disparities in aforementioned analyses. No statistically significant differences were detected between males and females (*t*-test with Bonferroni correction for multiple comparisons, *P* > 0.05) (Figure S3A and B), suggesting little genetic influence on the higher risk in men as observed above.

### LASSO-based MRS outperforms linear models and contributes to OrH risk profiling

**Figure 3:**
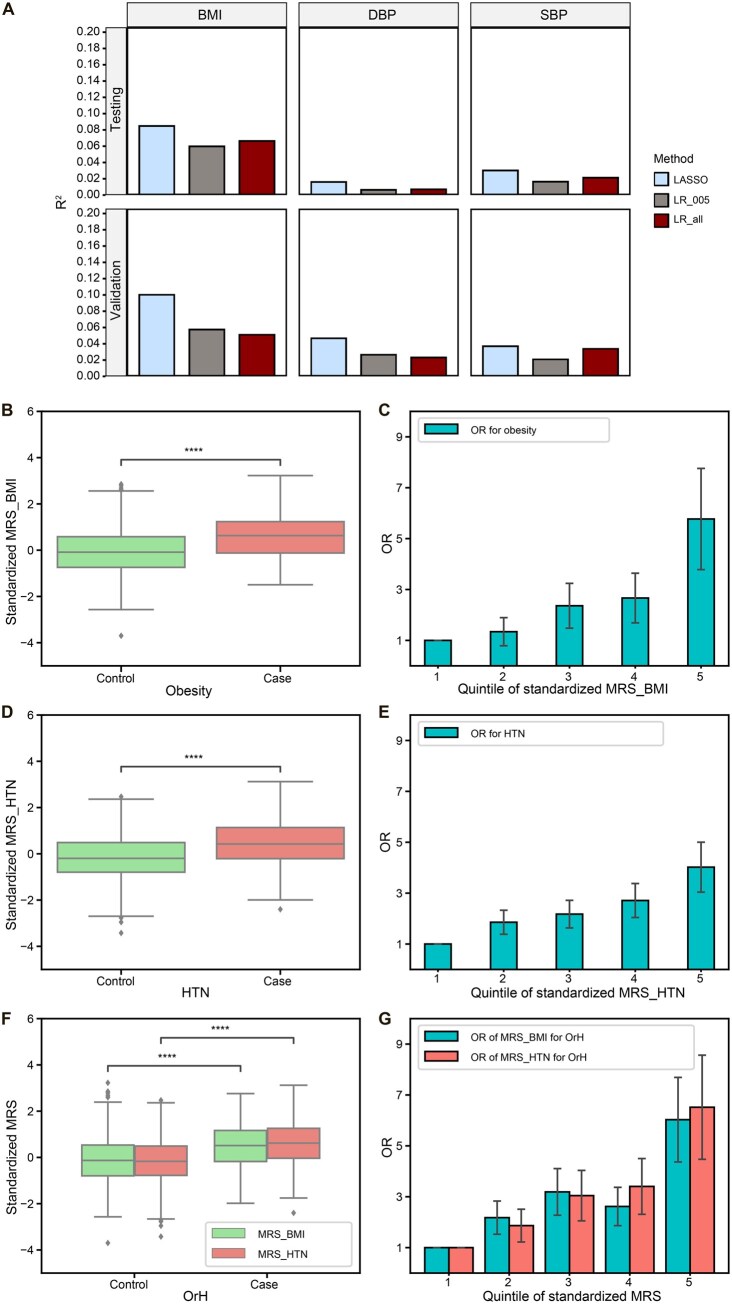
Performance of MRSs for BMI, DBP, and SBP as well as their associations with obesity, HTN, and OrH **A**. R^2^ values (Y-axis) represent the proportion of phenotypic variance explained by the MRSs for BMI, DBP, and SBP across different methods (X-axis). Results are shown for the testing set (*n* = 1466, upper) and the validation set (*n* = 1071, lower). Each bar corresponds to a specific method, as indicated by the color-coded legend. The phenotype was regressed on age, sex, and cell composition, and the residual from this regression was used as the dependent variable in the MRS modeling analyses. **B**. Boxplot of MRS for BMI (MRS_BMI) in control *vs*. obesity case groups. **C**. OR for obesity across quintiles of MRS_BMI, with the lowest 20% quintile serving as the reference group. **D**. Boxplot of MRS for HTN (MRS_HTN) in control *vs*. HTN case groups. **E**. OR for HTN across quintiles of MRS_HTN, with the lowest 20% quintile serving as the reference group. **F**. Boxplot of MRS for BMI (MRS_BMI) and HTN (MRS_HTN) in control *vs*. OrH case groups. **G**. OR for OrH across quintiles of MRS_BMI and MRS_HTN, with the lowest 20% quintile serving as the reference group. ****, *P* < 0.0001 (Wald test). MRS, methylation risk score; LASSO, least absolute shrinkage and selection operator; LR_005, linear regression using only significant CpG sites with *P* < 0.05; LR_all, linear regression using all available CpG sites.

Three MRS methods [LASSO, linear regression 1, and linear regression 2] were compared using distinct subsets of the NSPT cohort for MRS tuning (*n* = 2047, NSPT phase 3) and testing (*n* = 1466, NSPT phase 1 + phase 2), as well as the validation set (*n* = 1071, CAS1k) for final evaluation (see Materials and methods). We focused on 1506 CpG sites for BMI, 77 for DBP, and 107 for SBP by reviewing prior EWAS results (Table S11) [10–22]. All MRSs were normally distributed (Kolmogorov–Smirnov normality test with Bonferroni correction for multiple comparisons, *P* > 0.05). Among the three methods, LASSO achieved the best performance across all phenotypes in the MRS testing set ($R_{\mathrm{BMI}}^{2}=8.48\%$, $R_{\mathrm{DBP}}^{2}=1.61\%$, and $R_{\mathrm{SBP}}^{2}=3.02\%$,) and the validation set ($R_{\mathrm{BMI}}^{2}=10.03\%$, $R_{\mathrm{DBP}}^{2}=4.68\%$, and $R_{\mathrm{SBP}}^{2}=3.70\%$) (**Figure 3**A; Table S15).

When compared to the baseline models that included sex and age as predictors, the LASSO MRS showcased enhanced accuracy in the validation set. Specifically, R^2^ values increased from 18.28% to 26.86% for BMI, from 17.86% to 23.15% for DBP, and from 20.47% to 25.72% for SBP (Table 2). Notably, MRS_BMI exhibited a substantial difference between obesity and non-obesity individuals (*P* = 6.09E−11) (Figure 3B) and an OR/SD of 1.88 for obesity (95% CI: 1.57–2.24, *P* = 4.94E−09) (Table S14). In a five-quantile schema, the OR for obesity rose progressively across quintiles, reaching 5.77 in the highest quintile (Figure 3C; Table S14). This integration improved the AUC for obesity from 0.55 to 0.70 (Table 2). Similarly, MRS_HTN revealed a clear differentiation between the HTN and non-HTN groups (*P* = 6.87E−21) (Figure 3D), with an OR/SD of 1.65 (95% CI: 1.43–1.89, *P* = 3.85E−09) (Table S14). The ORs for HTN increased gradually across the five quantiles, peaking at 4.02 in the highest quintile (Figure 3E; Table S14). This integration improved the AUC for HTN from 0.70 to 0.73 (Table 2). When considering both MRS_BMI and MRS_HTN in the OrH model, there was an increase in AUC from 0.71 to 0.78 (Table 2). Both MRS_BMI and MRS_HTN demonstrated significant differentiation between the OrH and non-OrH groups (*P* = 7.17E−16 and *P* = 3.83E−23, respectively) (Figure 3F), with an OR/SD of 1.52 in MRS_BMI (95% CI: 1.31–1.77, *P* = 4.42E−06) (Table S14) and 1.72 in MRS_HTN (95% CI: 1.46–2.02, *P* = 4.99E−08) (Table S14). In the highest quintile, the ORs for OrH peaked at 6.03 and 6.52 for MRS_BMI and MRS_HTN, respectively (Figure 3G; Table S14).

Notably, males exhibited significantly higher MRSs than females for BMI and blood pressure (1.20E−41 < *P <* 5.08E−04) (Figure S3). These disparities may suggest the distinct influence of life styles and environmental exposures between genders in this cohort.

### Impact of metabolic and lifestyle factors on MRS predictions

We conducted a grouping analysis in the validation set based on MRS prediction errors to assess whether other metabolic and lifestyle factors were associated with discrepancies between MRS-predicted and observed values of BMI, DBP, and SBP (Table S16). The results indicate that discrepancies between the predicted and observed values are indeed associated with specific metabolic and lifestyle factors. For BMI, participants in the underestimated group (the lowest 10% quantile of prediction errors) tended to have healthier lipid profiles (higher HDL, lower TG, and lower LDL) and better lifestyle scores. Conversely, the overestimated group (the highest 10% quantile) was associated with less favorable lipid profiles, higher FBG, and poorer lifestyle scores. Similar patterns were observed for DBP and SBP, where overestimation by the MRS was linked to higher levels of TC, LDL, and FBG, as well as lower lifestyle scores. These results indicate that metabolic health and lifestyle behaviors may influence the accuracy of MRS predictions for these cardiovascular risk factors.

### Multi-omics model improves OrH risk profiling

**Figure 4:**
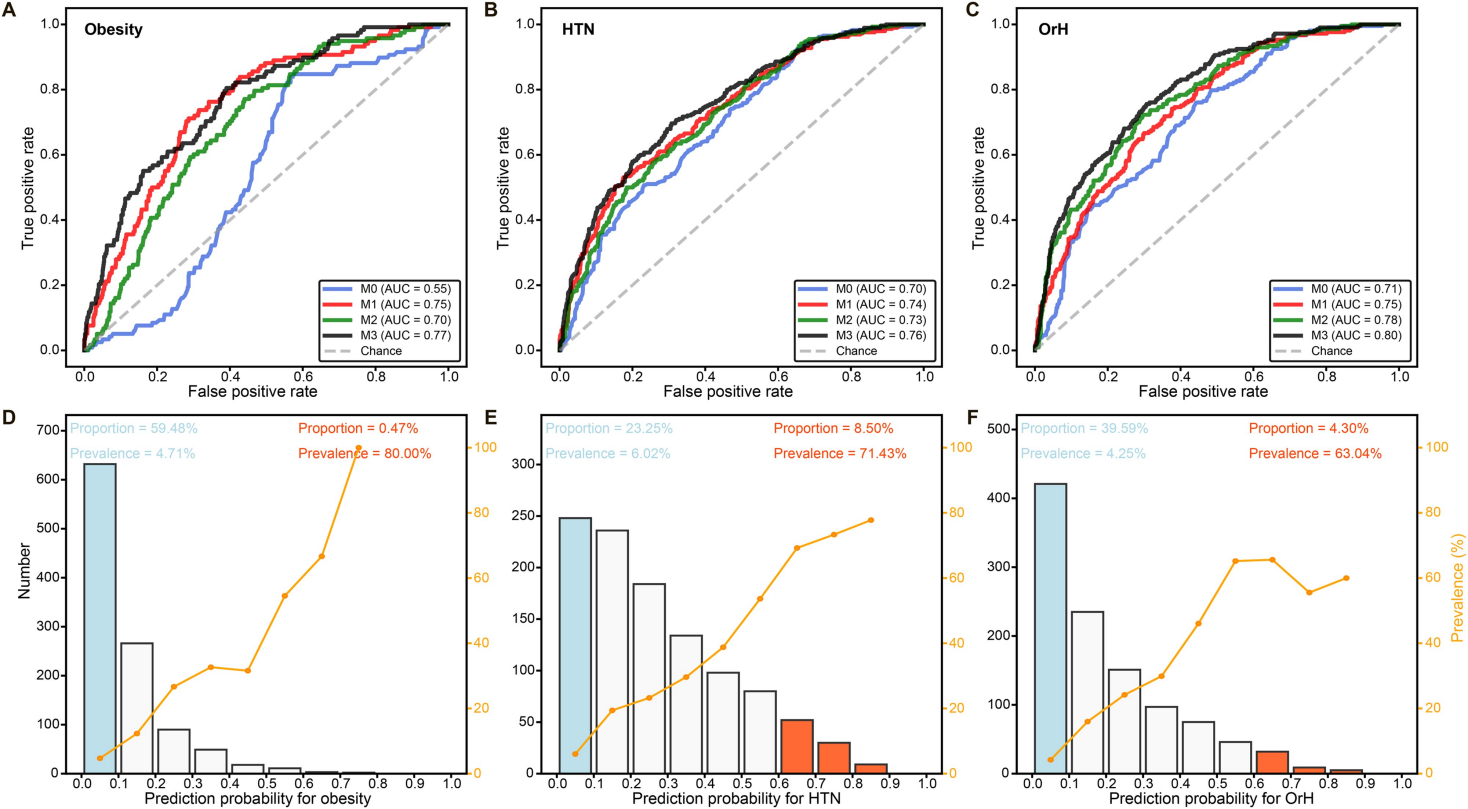
Prediction performance of multi-omics models for obesity, HTN, and OrH in the validation set **A**.–**C**. Assessment of AUC values for obesity (A), HTN (B), and OrH (C) using a 5-fold cross-validated logistic regression model. Predictors included age and sex (M0), age, sex, and PRS (M1), age, sex, and MRS (M2), and age, sex, PRS, and MRS (M3) in the validation set (*n* = 1071). **D**.–**F**. In the multi-omics prediction model (M3), the analyses further focused on individuals with extreme prediction probabilities for obesity (D), HTN (E), and OrH (F). Bars represent the number of participants within specific prediction probability intervals, with blue bars indicating low risk (< 0.10) and red bars indicating high risk (> 0.60). The orange line represents the prevalence in each interval. AUC, area under the curve.

We further integrated MRS and PRS into a multi-omics score and assessed its performance in predicting the risk of obesity, HTN, and OrH in the validation set according to a five-fold cross-validation design. For obesity, integrating MRS_BMI into the PRS model improved the AUC from 0.75 to 0.77 (**Figure 4**A; Table 2). This multi-omics score fairly classified 0.47% of the population as high risk (prediction probability > 0.6), who indeed showed a high prevalence of 80.00% (Figure 4D); meanwhile, it effectively identified 59.48% of the population as low risk (prediction probability < 0.1), who in fact had a low prevalence of 4.71%. Consequently, this indicates that our model is informative for 59.95% of the population in obesity risk profiling. For HTN, integrating MRS_SBP and MRS_DBP into the PRS model improved the AUC from 0.74 to 0.76 (Figure 4B; Table 2). This model is informative for 31.75% of the population in HTN risk profiling, effectively classifying the high-risk group (8.50% with prediction probability > 0.6 and a prevalence of 71.43%) and low-risk group (23.25% with prediction probability < 0.1 and a prevalence of 6.02%) (Figure 4E). In particular, for OrH, integrating MRS_BMI, MRS_SBP, and MRS_DBP into the PRS model boosted the AUC from 0.75 to 0.80 (Figure 4C; Table 2). This is informative for 43.89% of the population in OrH risk profiling, effectively classifying the high-risk group (4.30% with prediction probability > 0.6 and a prevalence of 63.04%) and low-risk group (39.59% with prediction probability < 0.1 and a prevalence of 4.25%) (Figure 4F).

## Discussion

In this study, we aimed to develop an accurate and effective prediction model for OrH by analyzing data from two general population cohorts, CAS and NSPT, totaling 7605 individuals. We assessed the performance of ten methods for PRSs and three strategies for MRSs using a tuning–testing–validation approach. Additionally, we developed a multi-omics model to enhance prediction accuracy. Throughout our analysis, we also found distinct population characteristics among academics in our study.

### Factors related to gender disparity in disease prevalence among Chinese academics

One unexpected observation in our study was the notably high prevalence of obesity, HTN, and OrH among males in Chinese academics. Therefore, we reviewed epidemiological data from the past three decades, which show significant changes in the prevalence of these conditions [45–50], most likely driven by China’s rapid economic growth and various risk factors, including economic status, gender, age, education, smoking, drinking, and inhabiting regions [51–56]. The M/F ratio for obesity has fluctuated between 0.6 and 1.3 in both urban and rural areas, with a marked disparity in urban areas (M/F ratio = 1.9) and even more pronounced among academics (M/F ratio = 3.8) (Table S3). For HTN, the M/F ratio in the CAS cohort reaches 2.9, significantly higher than in the NSPT cohort and broader Chinese epidemiological studies, where it ranges from 1.1 to 1.3.

The gender disparity in disease prevalence observed among Chinese academics aligns with findings from several studies. Research from the China Health and Nutrition Survey and the Chinese Center for Disease Control and Prevention has indicated that women with higher education levels tend to have a lower BMI and reduced odds of being overweight, while men with higher education levels exhibit a higher BMI and increased odds of being overweight in China [57,58]. Similar patterns have been observed in studies from Brazil, Russia, India, China, South Africa (BRICS) economies [59] and Southern European countries [60,61], further highlighting the association between education levels and obesity, particularly among women. Some studies have also examined the impact of education on HTN, indicating that individuals with higher education levels generally have healthier blood pressure. A Mendelian randomization study using data from FinnGen and the UKB suggests a causal relationship between education level and HTN. For each SD increase in genetically predicted higher education, the risk of HTN decreases by 44% [62]. Additionally, a study involving approximately 1.28 million adults from the China Health Evaluation And risk Reduction through nationwide Teamwork (ChinaHEART) project has found that as education level increases, there is a significant downward trend in SBP [63].

To explore the potential reasons for the significant gender disparity, we first examined the genetic possibility and found no significant genetic differences in the performance of PRS. However, notable epigenetic differences were observed in MRS for the corresponding diseases between males and females. Consistent with previous studies linking higher MRS to poorer metabolic health [11,64], our analysis showed that male academics exhibited higher MRS on BMI, DBP, and SBP, along with their relatively unhealthy lifestyles and metabolic syndrome traits. These findings suggest that the observed gender disparity is likely influenced by the combined effects of metabolic health and epigenetic factors.

We then briefly explored potential epigenetic explanations for the observed gender differences in disease prevalence. First, when examining regular physical activity, only minimal differences between sexes were found (Table S5), which aligns with data of a 15-year national survey [65]. Thus, physical activity does not provide a compelling explanation for the gender disparities observed in the CAS cohort. However, for other lifestyle factors and metabolic syndrome profiles, male academics showed significantly poorer parameters compared to their female counterparts (Tables S4 and S5). These differences are likely related to the more frequent social gatherings in males nationwide, such as dinners and drinking events, which are commonly associated with higher calorie expenditure and alcohol consumption [66]. Additionally, men may experience great social pressure as the primary bread-winners for their families, which is also likely associated with unhealthy lifestyle choices and an increased risk of metabolic-related diseases [67,68]. Considering the dominant male composition in academia (especially in full professors), one possible reason for the notably high M/F ratio (3.8) in the CAS cohort could be the intense academic pressure and heavy workload in males, which may largely boost unhealthy lifestyle habits. On the other hand, cultural attitudes in China tend to favor slimmer figures for women [47], and female academics may possess greater self-discipline regarding their health as well as more resources and opportunities to maintain their body shapes [55], which further accentuate the gender disparity observed in our cohort.

### Leveraging a multi-omics approach to enhance OrH risk prediction

OrH, as a common comorbidity pattern, would exacerbate cardiovascular and cerebrovascular damage more aggressively than simple obesity and HTN. Especially, its prevalence has shown a significant upward trend globally in the past 20 years. Although the integration of PRS and MRS demonstrates improved utility in various diseases, multi-omics prediction models for OrH remain limited. In this study, we developed such an approach for OrH prediction using both genomic and epigenomic signals, achieving an AUC as high as 0.80.

We first developed effective PRSs for the CAS cohort to predict BMI, DBP, and SBP by benchmarking five single-ancestry approaches (C+T, SCT, PRS-CS, LDpred2, and lassosum) and five multi-ancestry approaches (PRS-CSx, CT-SLEB, PolyPred-P+, JointPRS, and PROSPER). Among all methods, the top 3 with the highest accuracy are multi-ancestry methods across all traits. For BMI, the R^2^ values for the top 3 multi-ancestry methods ranged from 8.74% to 9.81%, compared to the best single-ancestry model, which achieved 6.36% in validation analysis (Table S12). Similarly, for DBP, the highest R^2^ of multi-ancestry models was 5.18%, surpassing the best single-ancestry model of 3.28%. Our results well confirm the outperformance of multi-ancestry PRS approaches over single-ancestry, and further demonstrate the enhanced generalizability of multi-ancestry PRS by leveraging shared genetic effects across different ancestries.

Among multi-ancestry PRS models, PRS-CSx consistently exhibited robust performance, achieving the highest R^2^ for BMI and consistently ranking among the top 3 models for both DBP and SBP in the validation set. This superior performance of complex traits across ancestries may be attributed to PRS-CSx’s advantage of Bayesian continuous shrinkage. Except for being only slightly (< 0.4%) behind PGS003882 and PGS005015 for SBP in the validation set, the PRS-CSx model developed in our study notably outperforms many published models in the PGS Catalog (116 PRSs for BMI, 72 for SBP, and 51 for DBP). These results further emphasize the potential of our PRS profiling for the Chinese population as a robust and reliable tool for genetic risk prediction and precision medicine applications.

Unlike genetic models, methylation data provide a real-time snapshot of an individual’s risk profile by capturing the epigenetic landscape, which reflects not only genetic susceptibility but also modifiable influences that contribute to disease progression, as reported in numerous studies [69,70]. Therefore, we aimed to use MRS for potential disease prediction based on currently available baseline data. After feature selection and optimization across various methylation models, LASSO-based MRS demonstrated the best performance in predicting BMI, DBP, and SBP, achieving similar or better R^2^ values compared to previous studies (*e.g.*, 10.03% for BMI *vs*. 10.00% reported [29], and 4.68% for blood pressure *vs*. 3.99% reported [28]). Considering the environmental or lifestyle factors, these MRS models, especially with longitudinal data in the future, may provide valuable insights into an individual’s health status and potentially serve as early warnings for unhealthy conditions.

Moreover, combining MRS with PRS enhances risk prediction by linking genetic susceptibility with current epigenetic states. Indeed, compared to the AUC of 0.75 for PRS and 0.78 for MRS, we observed an integrated AUC of 0.80 for OrH risk profiling, further confirming a shared molecular mechanism in obesity and HTN. This profiling may also fill a critical gap in individualized early warning for cardiovascular and metabolic disorders. By identifying high-risk individuals (risk score > 0.6) using multi-omics models, such as 4.30% for OrH in the CAS cohort, healthcare providers can implement more targeted preventive measures and treatment strategies to improve their health status.

## Conclusion

This research reveals a notably high prevalence of obesity, HTN, and OrH among males but significantly lower prevalence among females in Chinese academics with characterizations of research career and higher education. These results considerably diverge from common patterns observed in Chinese epidemiological investigations. Additional analysis indicates such large gender disparities are primarily associated to the complex interplay among epigenetic factors, lifestyle, and metabolic health, raising concerns about notably higher risks for males within Chinese academics. In omics analysis, PRS-CSx and LASSO-based MRS demonstrate high potential as robust tools for risk assessment of obesity, HTN, and OrH. The integration of PRS and MRS further enhance the accuracy of the risk profiling, suggesting the effectiveness of multi-omics approaches for improved personalized risk assessment strategies especially for OrH high-risk populations.

## Materials and methods

### Study population

#### The CAS cohort

This study involved 4092 Chinese participants from the CAS cohort, which was established in 2015 to target employees of the CAS in Beijing, China. The cohort was highly educated, with 99.4% holding at least a university degree. Participants first completed an online questionnaire that gathered information on factors such as gender, smoking status, alcohol consumption, tea intake, and sleep duration. Subsequently, they underwent clinical assessments at designated hospitals, including anthropometric, physical, blood, urine, and imaging exams, with 8 ml of blood collected from each participant.

Recruitment occurred in two phases. Phase 1 (2016–2018) included 991 participants; their DNA samples were analyzed using 30× whole-genome sequencing (WGS), and all phenotypic data were collected at the General Hospital of Aviation Industry Corporation of China. Phase 2 (2020–2021) added 3101 participants; their DNA samples were analyzed using Illumina genotyping microarrays, and all phenotypic data were collected at Beijing Zhongguancun Hospital. In phase 2, a subset of 1071 individuals (the CAS1k subgroup) was designed to provide multi-omics data, with their samples further analyzed using Illumina methylation microarrays.

#### The NSPT cohort

The NSPT cohort is a population-based prospective cohort study consisting of 3523 Han Chinese individuals from multiple regions in China, including Taizhou, Nanning, and Zhengzhou (1310 males and 2213 females; aged from 18 to 83 years, mean ± SD = 50.21 ± 12.75). After quality control, 3513 participants were retained for subsequent analysis. Recruitment occurred in three phases: phase 1 (*n* = 690) in 2018, phase 2 (*n* = 776) in 2019, and phase 3 (*n* = 2047) in 2019. DNA methylation was assessed using Illumina methylation microarrays on blood samples.

### Definitions of overweight, obesity, HTN, OrH, healthy lifestyle, and higher education

Overweight was defined as a BMI between 24.0 and 27.9 kg/m^2^, while obesity was defined as BMI ≥ 28.0 kg/m^2^, according to China’s guidelines [71]. HTN was defined as SBP ≥ 140 mmHg, DBP ≥ 90 mmHg, self-reported HTN diagnosis, or use of antihypertensive medications. Individuals with both BMI ≥ 24.0 kg/m^2^ and HTN were categorized as having OrH [72]. It is important to note that thresholds for defining obesity or HTN may vary across populations [73,74], and comparisons with studies using different criteria should be interpreted with caution.

Healthy lifestyle factors were defined based on the China Kadoorie Biobank (CKB) criteria [75], which include non-smoking, no excessive alcohol consumption, healthy diet (daily fruit and vegetable intake), regular physical activity, normal BMI (18.5–23.9 kg/m^2^), and normal WC (< 85 cm for males and < 80 cm for females). Participants earned a score of 1 for each criterion that they met and 0 for each one that they did not, resulting in a total score ranging from 0 to 6, representing their overall healthy lifestyle. Higher education was defined as having any college or university degree.

### WGS and microarray genotyping in the CAS cohort

WGS was performed at 30× coverage on the Illumina HiSeq 3000 platform (Illumina, San Diego, CA), and sequencing reads were aligned to the hg19 reference genome [76]. Variants were called using Genome Analysis Toolkit (GATK) [77] and annotated using ANNOVAR [78], with detailed methods for sample and library preparation reported previously [79].

Microarray genotyping was conducted on the Infinium Asian Screening Array + MultiDisease-24 BeadChip (Illumina, San Diego, CA). SNP genotypes were phased and imputed using IMPUTE2 [80] based on the East Asian population in the 1000 Genomes Project [81,82].

Quality control included removing individuals with gender mismatch, low genotyping call rate (< 97%), or abnormal heterozygosity (outside the mean ± 3 SD range). For SNPs, we excluded those with imputation score < 0.6 (in the CAS phase 2 cohort), Hardy–Weinberg equilibrium *P* < 1E−04, genotyping call rate < 98%, and minor allele frequency (MAF) < 1%. After these steps, 3,169,262 SNPs and 4092 individuals were retained for subsequent analysis.

### Methylation microarray of the CAS1k and NSPT cohorts

DNA methylation profiling for both the CAS1k and NSPT cohorts was performed on the Illumina Infinium MethylationEPIC BeadChip (Illumina, San Diego, CA). The raw array data were processed using the ChAMP package [83] in R to compute β values for methylation levels. Probes were filtered based on Illumina quality thresholds (bead count < 3 in > 5% of samples and 1% of samples with a detection *P* value > 0.05). Batch effects were corrected using ComBat [84,85], and cell-type heterogeneity was adjusted using EpiDISH [86]. After quality control, 751,015 CpG sites were retained for the CAS1k cohort, and 811,876 CpG sites were retained for the NSPT cohort.

### Construction and selection of PRSs

PRS construction and selection followed a tuning–testing–validation design using the CAS cohort. The tuning set included 2030 participants from phase 2 (excluding CAS1k), the testing set had 991 participants from phase 1, and the validation set consisted of 1071 participants from phase 2 (*i.e.*, the CAS1k cohort).

PRSs for BMI, DBP, and SBP were constructed using GWAS summary statistics from the UKB (∼ 450,000 Europeans) [87] and the BBJ (∼ 150,000 Japanese) [88,89]. Ten PRS methods were applied: C+T [34], SCT [35], PRS-CS [36], LDpred2 [37,38], lassosum [39], PRS-CSx [40], CT-SLEB [41], PolyPred-P+ [42], JointPRS [43], and PROSPER [44]. Hyperparameters were fine-tuned in the tuning set and evaluated in the testing and validation sets, with performance assessed by R^2^ and 95% CI using bootstrap resampling (*k* = 10,000, detailed in File S1).

To identify the optimal PRS for East Asians, the PRSs constructed by the ten methods were compared to existing scores in the PGS Catalog, selecting 48, 4, and 20 PRSs for BMI, DBP, and SBP, respectively, based on the required information. The best-performing PRS was then used for multi-omics prediction analysis in the validation set. In all PRS analyses, phenotypes were regressed on age, sex, and six genomic principal components, with the residuals used for PRS modeling to calculate adjusted R^2^ values reflecting variance explained beyond potential confounders.

### Construction and selection of MRSs

MRS construction and selection followed a tuning–testing–validation design using both the NSPT and CAS1k cohorts. The MRS tuning set included 2047 participants from phase 3 of the NSPT cohort, while the MRS testing set consisted of 1466 participants from the phase 1 and phase 2 of the NSPT cohort. The validation set was the CAS1k cohort (*n* = 1071). MRSs for BMI, DBP, and SBP were derived from findings in previous studies [10–22], resulting in final sets of 1506, 77, and 107 CpG sites for BMI, DBP, and SBP, respectively (details provided in File S1).

To construct the MRSs, we used three different methods: linear regression 1, which included all CpG sites from the studies without filtering; linear regression 2, which selected only CpG sites with a *P* value < 0.05; and LASSO regression, which applied penalized linear regression to optimize the model. Different CpG sets with corresponding beta coefficients were generated in the tuning set, then evaluated and validated in the testing and validation sets.

The best-performing MRS was selected and applied to the validation set for multi-omics prediction analysis. In all MRS analyses, phenotypes were regressed on age, sex, and cell composition, with the residuals serving as the dependent variable for MRS modeling. This approach enabled us to report adjusted R^2^ values and 95% CI, calculated through bootstrap resampling (*k* = 10,000), that account for the variance in MRS explained beyond the confounding effects of age, sex, and cell composition.

### Grouping analysis of MRS-predicted values

A grouping analysis of MRS-predicted values was performed in the validation set to determine whether discrepancies between MRS-predicted and observed values for BMI, DBP, and SBP were associated with metabolic and lifestyle factors. Participants were grouped into three groups based on the quantiles of the prediction error (predicted minus observed): the lowest 10% quantile, where MRS significantly underestimated the trait; the middle 80% quantiles, where MRS predictions closely matched observed values; and the highest 10% quantile, where MRS significantly overestimated the trait. Metabolic factors (including HDL, LDL, TC, TG, FBG) and healthy lifestyle scores were then compared across these groups using independent *t*-tests to identify significant differences between the underestimated, accurately predicted, and overestimated groups.

### Multi-omics risk prediction

The best-performing PRS and MRS were combined into multi-omics scores, and their prediction performance was assessed in the validation set, using linear regression for continuous traits (BMI, SBP, and DBP) and logistic regression for binary traits (obesity, HTN, and OrH). These models were adjusted for age and sex as covariates. Performance was evaluated using a five-fold cross-validation design. In brief, models were trained on four folds and tested on the remaining one, and this process was repeated five times, each time using a different part of the data for testing. The R^2^ values for continuous traits and AUC values for binary traits were averaged across the five iterations to provide a more generalized estimate of the model’s prediction power.

Predictors were specified as follows. For continuous traits, separate models were developed using the corresponding PRS, MRS, or both (multi-omics) as predictors. For binary traits, obesity models used PRS_BMI, MRS_BMI, or both as predictors; HTN models incorporated the average of PRS_DBP and PRS_SBP, the average of MRS_DBP and MRS_SBP, or both averages as predictors; OrH models included PRS_BMI plus the average of PRS_DBP and PRS_SBP, MRS_BMI plus the average of MRS_DBP and MRS_SBP, or the combination of all four predictors.

This modeling strategy ensured a consistent and robust comparison of baseline (sex and age only), PRS, MRS, and multi-omics models. All analyses were performed using R (v4.0.3) and Python (v3.6.4).

## Ethical statement

This study was approved by the Ethics Committee of the Beijing Institute of Genomics, CAS (Approval Nos. 2015H023 and 2021H001), the Ethics Committee of Beijing Zhongguancun Hospital (Approval No. 20201229), the Ethics Committee of Fudan University (Approval No. 14117), and the Ethics Committee of Shanghai Institutes for Biological Sciences (Approval No. ER-SIBS-261410). All participants provided written informed consent for the use of their samples and data for medical research.

## Code availability

C+T and SCT are available at https://github.com/privefl/bigsnpr/tree/cef0482c3c87ff51b63f5f2b0c896c75717ab92d/vignettes. PRS-CS is available at https://github.com/getian107/PRScs. PRS-CSx is available at https://github.com/getian107/PRScsx. CT-SLEB is available at https://andrewhaoyu.github.io/CTSLEB/. JointPRS is available at https://github.com/LeqiXu/JointPRS. PROSPER is available at https://github.com/Jingning-Zhang/PROSPER. LDpred and lassosum are available at https://privefl.github.io/bigsnpr/articles/LDpred2.html. PolyPred-P+ is available at https://github.com/omerwe/polyfun. The code has also been submitted to BioCode at the National Genomics Data Center (NGDC), China National Center for Bioinformation (CNCB) (BioCode: BT007949), which is publicly accessible at https://ngdc.cncb.ac.cn/biocode/tools/BT007949.

## Supplementary Material

qzaf048_Supplementary_Data

## Data Availability

The raw sequencing data from the CAS cohort have been deposited in the Genome Sequence Archive [90] at the National Genomics Data Center (NGDC), China National Center for Bioinformation (CNCB) (GSA: CRA000631), and are publicly accessible at https://ngdc.cncb.ac.cn/gsa. The genome sequence has been deposited in the Genome Warehouse [91] at the NGDC, CNCB (GWH: GWHAAAS00000000), and is publicly accessible at http://bigd.big.ac.cn/gwh. Additionally, the methylation data from the CAS and NSPT cohorts have been deposited in the Open Archive for Miscellaneous Data [90] at the NGDC, CNCB (OMIX: OMIX004333 for CAS, OMIX00436 for NSPT), and are publicly accessible at https://ngdc.cncb.ac.cn/omix.

## CRediT author statement

**Yaning Zhang:** Conceptualization, Methodology, Formal analysis, Writing – original draft. **Qiwen Zheng:** Conceptualization, Visualization, Writing – review & editing. **Qili Qian:** Data curation, Formal analysis. **Na Yuan:** Data curation. **Tianzi Liu:** Data curation. **Xingjian Gao:** Data curation. **Xiu Fan:** Data curation. **Youkun Bi:** Data curation. **Guangju Ji:** Data curation. **Peilin Jia:** Data curation. **Sijia Wang:** Conceptualization, Supervision, Writing – review & editing. **Fan Liu:** Conceptualization, Supervision, Writing – review & editing. **Changqing Zeng:** Conceptualization, Supervision, Writing – review & editing. All authors have read and approved the final manuscript.
