# Supplementary material for: An Integrative Polygenic and Epigenetic Risk Score for Overweight-related Hypertension in Chinese Population
Source: Genomics Proteomics Bioinformatics. 2025 Jun 16;23(5):qzaf048. doi: 10.1093/gpbjnl/qzaf048 (PMC12854722; doi:10.1093/gpbjnl/qzaf048)
Supplement: qzaf048_Supplementary_Data [file qzaf048_supplementary_data.zip › File S1.docx]

**Supplementary methods**

**UK Biobank and BioBank Japan genome-wide association study summary statistics**

In order to generate different ancestry polygenic risk scores (PRSs), we collected genome-wide association study (GWAS) summary statistics through previous studies of East Asian (EAS) and European (EUR) separately to be associated with body mass index (BMI), diastolic blood pressure (DBP), and systolic blood pressure (SBP). EUR GWAS data was collected from UK biobank (UKB) [1] while EAS GWAS data was collected from BioBank Japan (BBJ) [2,3]. UKB is a large-scale prospective cohort composed of about 500,000 of mainly EUR ancestry adults, whereas BBJ is a hospital-based disease-ascertained cohort with approximately 200,000 participants of mainly Japanese ancestry [4–6].

For the UKB GWAS summary, we included only autosomal single nucleotide polymorphisms (SNPs) with a minor allele frequency (MAF) greater than 1% and an INFO score exceeding 0.9, resulting in approximately 12 million SNPs for analysis. Similarly, for the BBJ GWAS summary, we considered autosomal SNPs with MAF > 1% and R^2^ > 0.9, yielding around 5.9 million SNPs for analysis. To ensure consistency in SNP magnitude across datasets, we performed analyses on the intersection of UKB GWAS summary, BBJ GWAS summary, and the Chinese Academy of Sciences (CAS) cohort. Following the correction of positive and negative strands in the different datasets, we identified 3,169,089 autosomal markers available for subsequent analyses. To facilitate uniformity in GWAS summary statistics, we reformatted the data to include SNP ID, chromosome, base pair position, effect allele (EA), non-effect allele (Non-EA), EA frequency, effect size estimate, effect size estimate standard error, and *P* value.

**Construction and selection of PRS**

To generate various PRS, we employed a total of 15 strategies using GWAS data from the UKB and BBJ for BMI, DBP, and SBP. The construction of PRS involved utilizing GWAS summary statistics from both the UKB or BBJ datasets. We employed 10 PRS construction methods, namely clumping and thresholding (C+T) [7], Stacked C+T (SCT) [8], PRS-CS [9], LDpred2 [10,11], lassosum [12], PRS-CSx [13], CT-SLEB [14], PolyPred-P+ [15], JointPRS [16], and PROSPER [17]. Hyperparameters for each method were fine-tuned within the PRS tuning set to determine the optimal PRS for each trait.

For single-ancestry PRSs, five methods (C+T, SCT, PRS-CS, LDpred2, and lassosum) were applied to either UKB or BBJ data, resulting in a total of 10 PRSs. For the optimization for C+T, we use the large grid of hyperparameters testing a threshold of clumping r^2^c within [0.01, 0.05, 0.1, 0.2, 0.5, 0.8, 0.95], a base size of clumping window within [50, 100, 200, 500] in kb where the actual window size is then computed as the base size divided by r^2^c, and a sequence of 20 thresholds on −log_10_(*P* values) between 0.1 and the most significant *P* value, equally spaced on a log scale. SCT PRS employed penalized regression to find the optimal linear combination of all C+T PRS. PRS-CS utilized a full Bayesian approach (a = 1, b = 0.5) to estimate the global shrinkage parameter, phi. For LDpred2, we tested 12 combinations of parameters, with *P* values selected from [0.003,0.01,0.03,0.1,0.3,1], h^2^ estimated from LDSC, and the sparse option set to [FALSE,TRUE]. For lassosum, we explored 80 parameter combinations using a large grid of hyperparameters, testing delta thresholds from [0.001,0.01,0.1,1], with nlambda set to 20 and lambda.min.ratio fixed at 0.01.

In the case of multi-ancestry PRSs, GWAS data from both UKB and BBJ were combined, and the PRS-CSx, CT-SLEB, PolyPred-P+, JointPRS, and PROSPER methods were applied, yielding five additional PRSs. Specifically, the PRS for PRS-CSx method for each phenotype integrated EAS and EUR GWAS summary statistics using the parameter, phi, a = 1, b = 0.5. For JointPRS, we used a linear combination of the PRSs with parameter (phi = auto, a = 1, b = 0.5) using EAS and EUR posterior SNP effect size estimates, taking the genetic correlation into consideration in addition. For CT-SLEB, we selected the best PRS from 2268 combinations using GWAS summary statistics from EAS and EUR, integrating C+T, empirical Bayes, and 3 super-learning models: glmnet, ridge, nnet. For PolyPred-P+, we combinated of PolyFun-pred with PRS-CS-UKB and PRS-CS-BBJ. And for PROSPER, PRS was developed using an ensemble of 50 combinations of delta, λ, and c, and 3 super-learning models: glmnet, SL.ridge, and lm.

A reference panel of 503 EUR individuals from the 1000 Genome Project was used for all methods when deriving effect sizes from the UKB GWAS. Additionally, 504 EAS individuals from the 1000 Genome Project served as the linkage disequilibrium (LD) reference for all methods when effect sizes were derived from the BBJ GWAS.

The optimized PRSs underwent evaluation in the PRS testing and validation set, assessing their performance through model fitness R^2^ and calculating the 95% confidence interval (CI) using bootstrap resampling (k = 10,000). In all PRS modeling testing procedures, the phenotype was regressed on age, sex, and six genomic principal components. The residuals from this regression were then used as the dependent variable in PRS modeling and testing analyses.

**CpG selection for MRS**

Numerous CpG sites have been associated with BMI due to the multifaceted nature of metabolic syndrome, which includes hyperlipidaemia, hyperglycaemia, and inflammatory changes reflected in DNA methylation changes as highlighted by Bell et al. [18] and Do and his colleagues [19]. We developed comprehensive lists of CpG sites for BMI, DBP, and SBP by systematically integrating findings from previous studies and applying rigorous quality control measures.

For BMI, we included 1109 CpGs identified by McCartney et al. [20], retaining 1075 after quality control. We further incorporated 397 CpGs reported by Do et al. [19], with 368 passing quality checks, along with 3 CpG sites discovered by Li and his colleagues [21]. And 2 CpGs identified by Dick et al. [22], 223 of 239 CpGs identified by Wahlet al. [23], and 2 CpGs from Chen et al. [24] were also included. After excluding overlapping CpGs across studies, the final list for BMI comprised 1506 unique CpGs.

For DBP, we included 42 of 47 CpGs from Richard et al. [25], 26 of 28 CpGs from Kato et al. [26], and 19 of 20 CpGs from Hong and his colleagues [27]. Following the removal of overlapping CpGs, the finalized list contained 77 unique CpGs for DBP.

For SBP, we incorporated 26 of 28 CpGs from Hoyt et al. [28], 1 CpG from Huan et al. [29], 2 CpGs from Si et al. [30], 16 of 17 CpGs from Hong et al. [27], 1 CpG from Kou et al. [31], and 73 of 77 CpGs from Richard and his colleagues [25]. After accounting for overlaps, the final list included 107 unique CpGs for SBP.

**Reference**

[1] Canela-Xandri O, Rawlik K, Tenesa A. An atlas of genetic associations in UK Biobank. Nat Genet 2018;50:1593–9.

[2] Kanai M, Akiyama M, Takahashi A, Matoba N, Momozawa Y, Ikeda M, et al. Genetic analysis of quantitative traits in the Japanese population links cell types to complex human diseases. Nat Genet 2018;50:390–400.

[3] Akiyama M, Okada Y, Kanai M, Takahashi A, Momozawa Y, Ikeda M, et al. Genome-wide association study identifies 112 new loci for body mass index in the Japanese population. Nat Genet 2017;49:1458–67.

[4] Nagai A, Hirata M, Kamatani Y, Muto K, Matsuda K, Kiyohara Y, et al. Overview of the BioBank Japan Project: study design and profile. J Epidemiol 2017;27:S2–8.

[5] Hirata M, Nagai A, Kamatani Y, Ninomiya T, Tamakoshi A, Yamagata Z, et al. Overview of BioBank Japan follow-up data in 32 diseases. J Epidemiol 2017;27:S22–8.

[6] Hirata M, Kamatani Y, Nagai A, Kiyohara Y, Ninomiya T, Tamakoshi A, et al. Cross-sectional analysis of BioBank Japan clinical data: a large cohort of 200,000 patients with 47 common diseases. J Epidemiol 2017;27:S9–21.

[7] Wray NR, Goddard ME, Visscher PM. Prediction of individual genetic risk to disease from genome-wide association studies. Genome Res 2007;17:1520–8.

[8] Privé F, Vilhjálmsson BJ, Aschard H, Blum MGB. Making the most of clumping and thresholding for polygenic scores. Am J Hum Genet 2019;105:1213–21.

[9] Ge T, Chen CY, Ni Y, Feng YA, Smoller JW. Polygenic prediction via Bayesian regression and continuous shrinkage priors. Nat Commun 2019;10:1776.

[10] Vilhjálmsson BJ, Yang J, Finucane HK, Gusev A, Lindström S, Ripke S, et al. Modeling linkage disequilibrium increases accuracy of polygenic risk scores. Am J Hum Genet 2015;97:576–92.

[11] Privé F, Arbel J, Vilhjálmsson BJ. LDpred2: better, faster, stronger. Bioinformatics 2021;36:5424–31.

[12] Mak TSH, Porsch RM, Choi SW, Zhou X, Sham PC. Polygenic scores via penalized regression on summary statistics. Genet Epidemiol 2017;41:469–80.

[13] Ruan Y, Lin YF, Feng YA, Chen CY, Lam M, Guo Z, et al. Improving polygenic prediction in ancestrally diverse populations. Nat Genet 2022;54:573–80.

[14] Zhang H, Zhan J, Jin J, Zhang J, Lu W, Zhao R, et al. A new method for multiancestry polygenic prediction improves performance across diverse populations. Nat Genet 2023;55:1757–68.

[15] Weissbrod O, Kanai M, Shi H, Gazal S, Peyrot WJ, Khera AV, et al. Leveraging fine-mapping and multipopulation training data to improve cross-population polygenic risk scores. Nat Genet 2022;54:450–8.

[16] Xu L, Zhou G, Jiang W, Zhang H, Dong Y, Guan L, et al. JointPRS: a data-adaptive framework for multi-population genetic risk prediction incorporating genetic correlation. Nat Commun 2025;16:3841.

[17] Zhang J, Zhan J, Jin J, Ma C, Zhao R, O’Connell J, et al. An ensemble penalized regression method for multi-ancestry polygenic risk prediction. Nat Commun 2024;15:3238.

[18] Bell CG. The epigenomic analysis of human obesity. Obesity (Silver Spring) 2017;25:1471–81.

[19] Do WL, Sun D, Meeks K, Dugué PA, Demerath E, Guan W, et al. Epigenome-wide meta-analysis of BMI in nine cohorts: examining the utility of epigenetically predicted BMI. Am J Hum Genet 2023;110:273–83.

[20] McCartney DL, Hillary RF, Stevenson AJ, Ritchie SJ, Walker RM, Zhang Q, et al. Epigenetic prediction of complex traits and death. Genome Biol 2018;19:136.

[21] Li W, Xia M, Zeng H, Lin H, Teschendorff AE, Gao X, et al. Longitudinal analysis of epigenome-wide DNA methylation reveals novel loci associated with BMI change in East Asians. Clin Epigenetics 2024;16:70.

[22] Dick KJ, Nelson CP, Tsaprouni L, Sandling JK, Aïssi D, Wahl S, et al. DNA methylation and body–mass index: a genome-wide analysis. Lancet 2014;383:1990–8.

[23] Wahl S, Drong A, Lehne B, Loh M, Scott WR, Kunze S, et al. Epigenome-wide association study of body mass index, and the adverse outcomes of adiposity. Nature 2017;541:81–6.

[24] Chen Y, Kassam I, Lau SH, Kooner JS, Wilson R, Peters A, et al. Impact of BMI and waist circumference on epigenome-wide DNA methylation and identification of epigenetic biomarkers in blood: an EWAS in multi-ethnic Asian individuals. Clin Epigenetics 2021;13:195.

[25] Richard MA, Huan T, Ligthart S, Gondalia R, Jhun MA, Brody JA, et al. DNA methylation analysis identifies loci for blood pressure regulation. Am J Hum Genet 2017;101:888–902.

[26] Kato N, Loh M, Takeuchi F, Verweij N, Wang X, Zhang W, et al. Trans-ancestry genome-wide association study identifies 12 genetic loci influencing blood pressure and implicates a role for DNA methylation. Nat Genet 2015;47:1282–93.

[27] Hong X, Miao K, Cao W, Lv J, Yu C, Huang T, et al. Association between DNA methylation and blood pressure: a 5-year longitudinal twin study. Hypertension 2023;80:169–81.

[28] Hoyt MF. Stepping into retirement: a postcard from the threshold. J Clin Psychol 2015;71:1121–7.

[29] Huan T, Joehanes R, Song C, Peng F, Guo Y, Mendelson M, et al. Genome-wide identification of DNA methylation QTLs in whole blood highlights pathways for cardiovascular disease. Nat Commun 2019;10:4267.

[30] Si J, Yang S, Sun D, Yu C, Guo Y, Lin Y, et al. Epigenome-wide analysis of DNA methylation and coronary heart disease: a nested case-control study. Elife 2021;10:e68671.

[31] Kou M, Li X, Shao X, Grundberg E, Wang X, Ma H, et al. DNA methylation of birthweight–blood pressure genes and changes of blood pressure in response to weight-loss diets in the POUNDS Lost trial. Hypertension 2023;80:1223–30.
