# Supplementary material for: An Integrative Polygenic and Epigenetic Risk Score for Overweight-related Hypertension in Chinese Population
Source: Genomics Proteomics Bioinformatics. 2025 Jun 16;23(5):qzaf048. doi: 10.1093/gpbjnl/qzaf048 (PMC12854722; doi:10.1093/gpbjnl/qzaf048)
Supplement: qzaf048_Supplementary_Data [file qzaf048_supplementary_data.zip › supplementary material captions.docx]

**Supplementary material**

**File S1 Supplementary methods**

**Figure S1 Manhattan plot of GWASs on BMI, DBP, and SBP from UKB and BBJ**

The *P* for each variant in the EAS (BBJ) and EUR (UKB) GWASs are depicted for BMI, DBP, and SBP. Chromosomal location is represented on the horizontal axis, and the negative log of the *P* is represented on the vertical axis. For each trait, the grey dashed lines indicate the most significant *P* in EAS GWAS and black dashed lines indicates the most significant *P* in EUR GWAS. EAS, East Asian; EUR, European; Chr, chromosome.

**Figure S2 Workflow of an integrative multi-omics model combining PRS and MRS for OrH risk prediction**

This study presents an integrative multi-omics model that combines PRS and MRS to enhance the profiling of OrH risk. Utilizing GWAS summary statistics from BBJ and the UKB, we systematically evaluated various PRS methodologies, including C+T, SCT, PRS-CS, LDpred2, lassosum, PRS-CSx, CT-SLEB, PolyPred-P+, JointPRS, and PROSPER. A Chinese cohort of 4092 individuals from the CAS was divided into three datasets: phase 2 (excluding CAS1k) for hyperparameter tuning (*n* = 2030), phase 1 for model testing (*n* = 991), and CAS1k for final validation (*n* = 1071). Additionally, we developed multiple MRS models (linear regression 1, linear regression 2, and LASSO regression) based on previous EWAS findings, using data from 3513 Chinese participants in the NSPT cohort. NSPT phase 3 was employed for MRS parameter tuning (*n* = 2047), while NSPT phases 1 and 2 served as testing sets (*n* = 1466) to evaluate MRS performance. The predictive efficacy of the MRS for OrH risk was further validated in the CAS1k validation set (*n* = 1071). After deriving the optimal parameters for linear models of PRS and MRS for BMI, SBP, and DBP, these scores were integrated into a multi-omics score. The performance of the integrated model in predicting binary outcomes (obesity, HTN, and OrH) was assessed in the CAS1k validation set (*n* = 1071) using a five-fold cross-validation approach. EWAS, epigenome-wide association studies; NSPT, the National Survey of Physical Traits.

**Figure S3 Sex-specific distributions of PRS and MRS in the validation set**

**A.** and **B.** The distributions of PRS for BMI (A) and HTN (B) (the average of PRS for DBP and PRS for SBP), respectively, with no significant difference in PRS for BMI between males and females (*P* = 1) and a marginally non-significant difference in PRS for HTN (*P* = 0.08). **C.** and **D**. The distributions of MRS for BMI (C) and HTN (D) (the average of MRS for DBP and MRS for SBP), respectively, highlighting statistically significant differences between Sexes for both MRS for BMI (*P* = 5.08E−04) and MRS for HTN (*P* = 1.20E−41). The analysis was conducted in the validation set (*n* = 1071), and the boxplots represent the median, interquartile range, and overall data distribution for each group.

**Table S1 Summary of BMI, SBP, and DBP associated SNPs from GWAS Catalog**

**Table S2 Summary of BMI, SBP, and DBP associated CpGs from published studies**

**Table S3 Summary of research on the prevalence of HTN and obesity in Chinese adults**

**Table S4 Comparison of metabolic syndrome indicators between males and females in CAS and NSPT cohort**

**Table S5 Comparison of lifestyle factors between males and females in validation set**

**Table S6 Description of the GWAS summary data for the PRS construction**

**Table S7 Fine tuning of the hyperparameters conducted for C+T in PRS tuning set**

**Table S8 Fine tuning of the hyperparameters conducted for LDpred2 in PRS tuning set**

**Table S9 Fine tuning of the hyperparameters conducted for lassosum in PRS tuning set**

**Table S10 Fine tuning of the hyperparameters conducted for CT-SLEB in PRS tuning set**

**Table S11 Number of SNPs or CpG sites used in different PRS and MRS methods for BMI, DBP, and SBP**

**Table S12 Performance of different PRS in predicting quantitative traits in tunning, testing, and validation sets**

**Table S13 The performance of PRSs in PGS Catalog**

**Table S14 ORs for Obesity, HTN, and OrH across PRS and MRS in the validation set**

**Table S15 Peformance of different MRS in tunning, testing, and validaltion sets**

**Table S16 Comparison of metabolic and lifestyle factors among participants stratified by prediction accuracy of MRS for BMI, DBP, and SBP**
