# Supplementary figures and images for: An Integrative Polygenic and Epigenetic Risk Score for Overweight-related Hypertension in Chinese Population
Source: Genomics Proteomics Bioinformatics. 2025 Jun 16;23(5):qzaf048. doi: 10.1093/gpbjnl/qzaf048 (PMC12854722; doi:10.1093/gpbjnl/qzaf048)

BMI EAS-GWAS

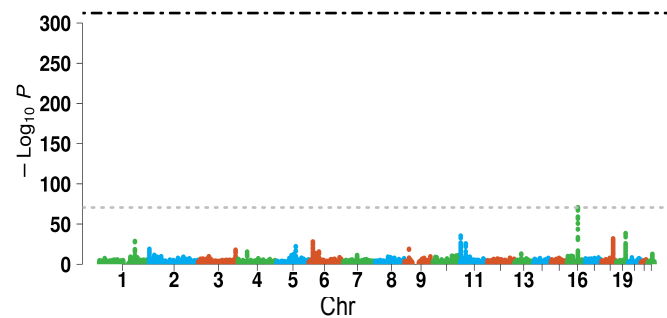

DBP EAS-GWAS

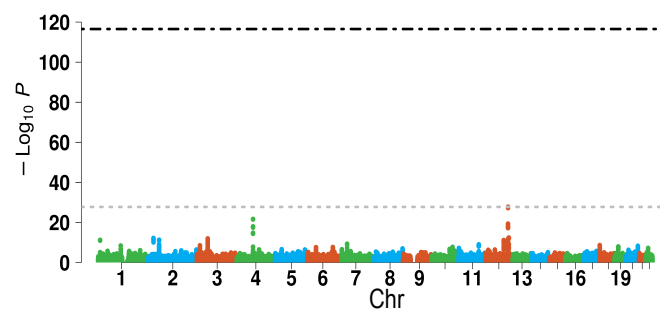

SBP EAS-GWAS

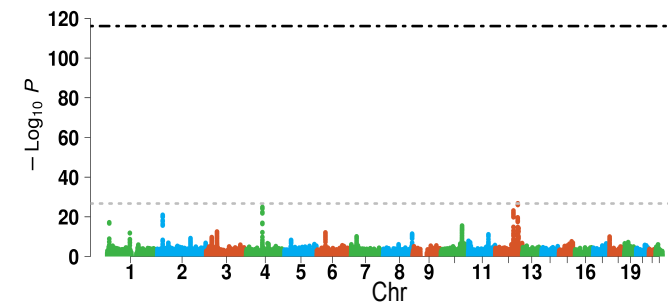

BMI EUR-GWAS

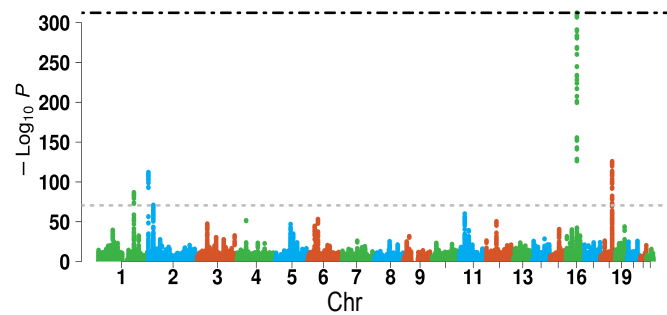

DBP EUR-GWAS

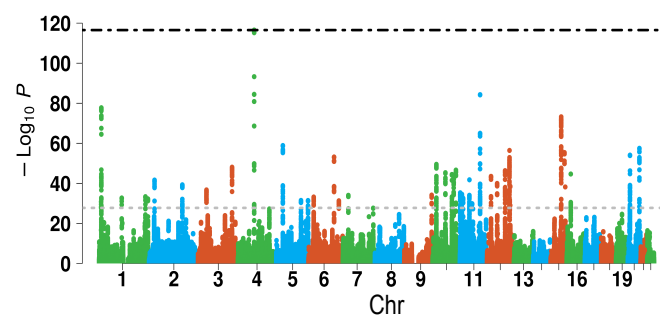

SBP EUR-GWAS

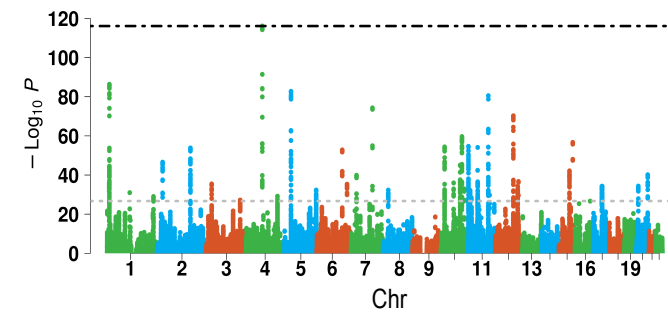

Supplement: qzaf048_Supplementary_Data [file qzaf048_supplementary_data.zip › Figure S1.pdf]

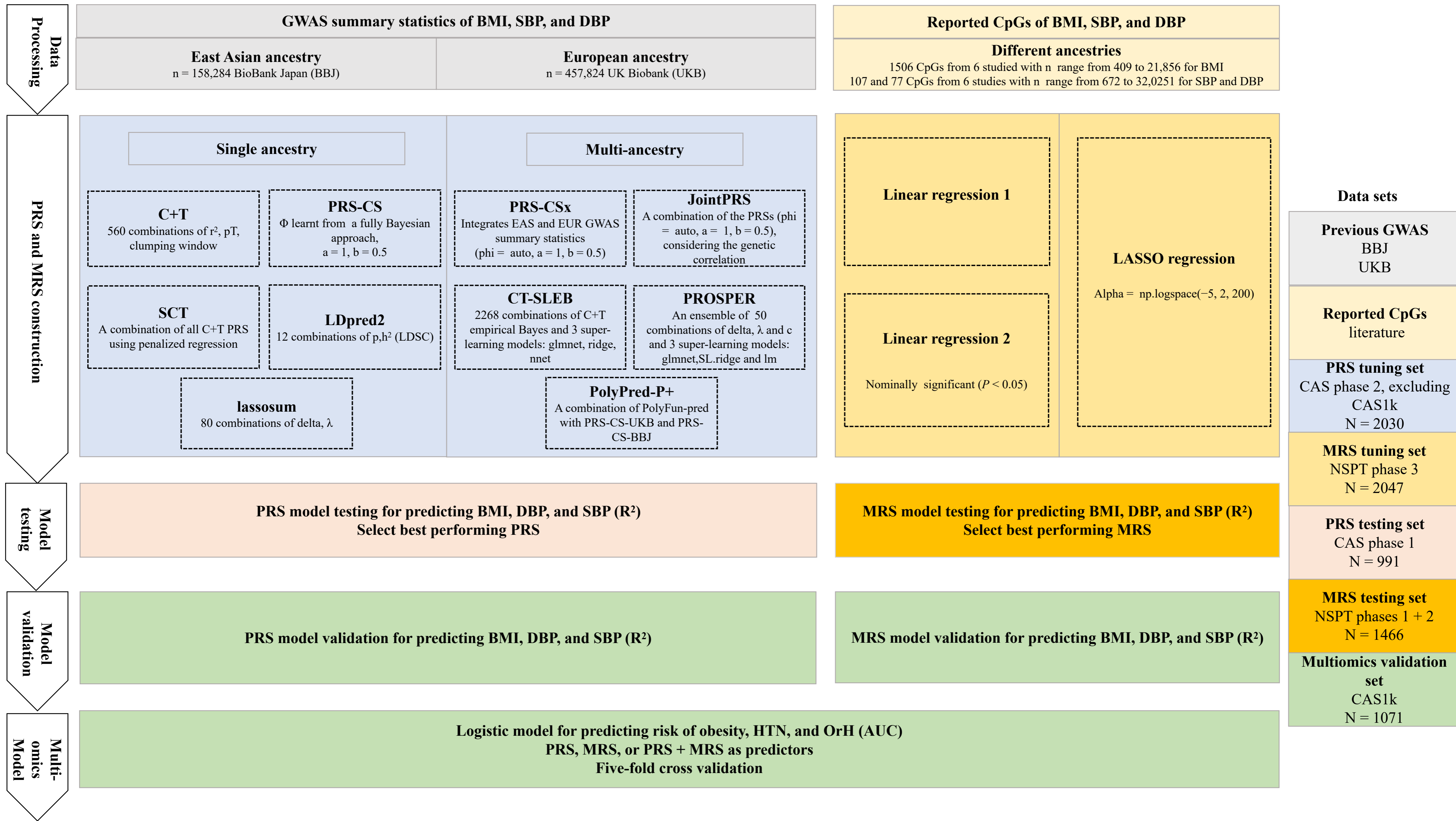

Supplement: qzaf048_Supplementary_Data [file qzaf048_supplementary_data.zip › Figure S2.pdf]

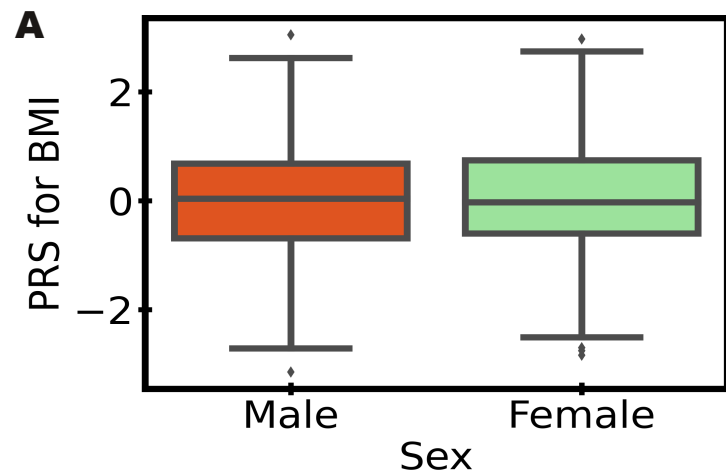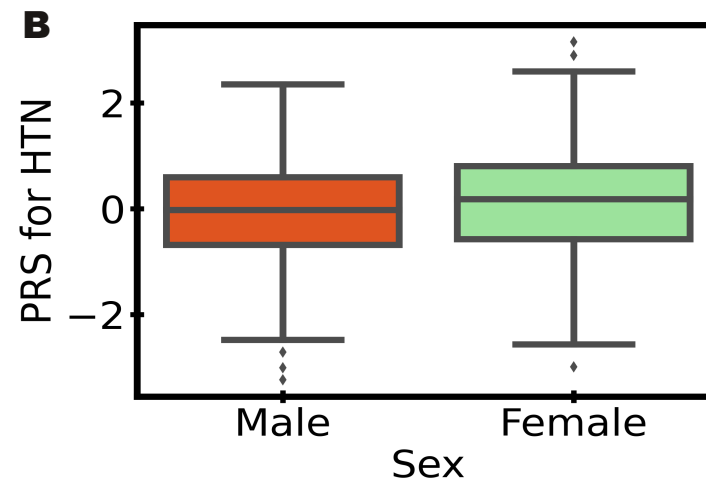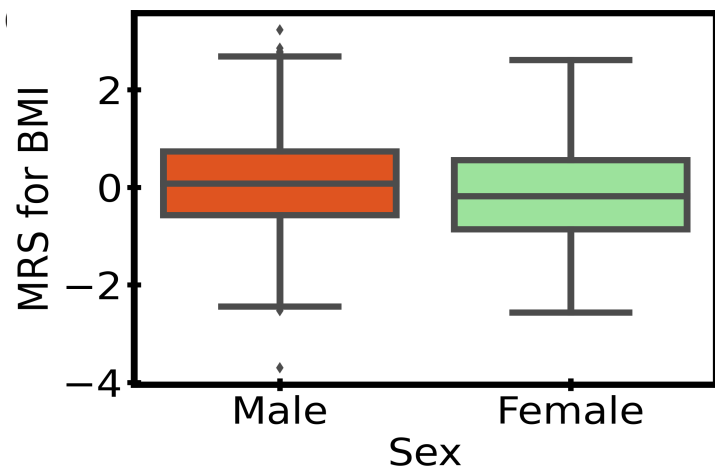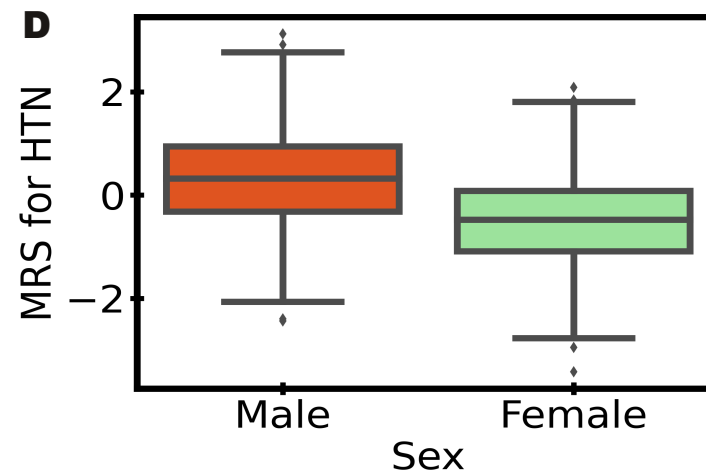

Supplement: qzaf048_Supplementary_Data [file qzaf048_supplementary_data.zip › Figure S3.pdf]
